# S10 Developing and Monitoring National Physical Activity Policy: A Global Challenge that Must be Addressed with a Global Response

**DOI:** 10.1093/eurpub/ckac093.049

**Published:** 2022-08-29

**Authors:** Andrea Ramirez Varela, Michael Pratt, Željko Pedišić, Adrian Bauman

**Affiliations:** 1 School of Medicine, Universidad de los Andes, Bogota, Colombia; 2 Institute for Public Health and MPH Program Department of Family Medicine and Public Health, University of California San Diego , San Diego, USA; 3 Institute for Health and Sport, Victoria University, Melbourne, Australia; 4 Sydney School of Public Health, University of Sydney, Sydney, Australia

**Keywords:** Physical activity, policy, national, global

## Abstract

**Goals:**

Describe the progress of developing and monitoring global physical activity policy from the 1990s through the release and implementation of Global Action Plan for Physical Activity-GAPPA;

Present findings of a systematic review of instruments for physical activity policy analysis;

Describe the methods and progress after 1-year implementation of the Global Observatory for Physical Activity-GoPA! physical activity policy inventory;

Present the preliminary findings and lessons learned after 1-year implementation of the Global Observatory for Physical Activity-GoPA! physical activity policy inventory.

**Issue/problem:**

Physical inactivity is one of the most important factors contributing to global morbidity and mortality, but despite both importance and the recent launch of a WHO action plan, public health policy around physical activity remains poorly developed and monitored.

Description of the problem

Physical inactivity accounts for as many as 5 million deaths per year globally but has yet to be addressed effectively by most governments or the World Health Organization(WHO). Many efforts led by WHO, national governments, networks, and academics from the 1990s to the present have tried to turn physical activity into a public health priority. Since its launch in 2015, the Global Observatory for Physical Activity-GoPA! has monitored the progress of national physical activity surveillance, policy and research. The GoPA! Policy Inventory created in 2017, contributes to physical activity policy monitoring and aims to collect data worldwide.

**Results:**

In this symposium we will address several key questions about physical activity policy by examining different initiatives such as GAPPA and the experience of monitoring policy globally with the GoPA! policy inventory. We will examine whether it is feasible to track physical activity policy at the global and country levels, if the existence of ?good? physical activity policy is associated with less physical inactivity and how policy indicators may be used for advocacy and guidance in the coming years.

**Lessons:**

Findings show that PA policy monitoring has and still varies substantially by geographic area and country income group. Effectiveness and implementation of PA policies remains challenging worldwide. PA policy indicators can enhance understanding of the links between policy and population levels of PA.

**Main messages:**

GoPA! is committed to continue improving PA policy monitoring for informing and encouraging policy to address physical inactivity worldwide.

Agenda and Presenters

Introduction: Michael Pratt (10-minutes)

Talk 1: Developing and monitoring physical activity policy: 1990 to today – Michael Pratt (15-minutes)

Talk 2: Instruments for the analysis of national-level physical activity and sedentary behaviour policies: a systematic review – Željko Pedišić (15-minutes)

Talk 3: The GoPA! Policy Inventory: Progress and methods - Andrea Ramirez (15-minutes)

Talk 4: The GoPA! Policy Inventory: preliminary findings and lessons learned – Željko Pedišić (15-minutes)

Q&A from the audience

Conclusion by discussant Adrian Bauman (20-minutes)

